# Evidence for inbreeding depression in captive Damaraland mole-rats

**DOI:** 10.1098/rsbl.2024.0407

**Published:** 2024-10-30

**Authors:** David Seager, Amy E. Leedale, Jack Benjamin Thorley, Philippe Vullioud, Markus Zöttl, Tim Clutton-Brock

**Affiliations:** ^1^ Department of Zoology, University of Cambridge, Cambridge, UK; ^2^ Kalahari Research Centre, Kuruman River Reserve, Northern Cape, South Africa; ^3^ School of Science, Engineering & Environment, University of Salford, Salford, UK; ^4^ Neuchâtel Platform of Analytical Chemistry, University of Neuchâtel, Neuchâtel, Switzerland; ^5^ Department of Biology and Environmental Science, Linnaeus University, Kalmar, Sweden; ^6^ Mammal Research Institute, University of Pretoria, Pretoria 0028, South Africa

**Keywords:** inbreeding, depression, cooperative, breeding

## Abstract

Mating between closely related individuals can result in a reduction in offspring fitness, known as inbreeding depression. Here, we investigate whether breeding with close relatives affects the reproductive output of parents and the development of their offspring in Damaraland mole-rats (*Fukomys damarensis*), a cooperatively breeding species where females avoid mating with familiar individuals. By cross-fostering litters of pups soon after birth, we were able to form breeding pairs from full siblings that were reared apart. We compared the reproductive output of these sibling pairs and the survival and growth of their pups with that of unrelated pairs over a period of 4 years. The litter sizes and interbirth intervals of sibling pairs did not differ from those of unrelated pairs, but the growth and survival of inbred offspring were lower, showing that breeding between close relatives is associated with substantial fitness costs. This study suggests that inbreeding depression is an important driver of the extreme reproductive skew observed in social mole-rats. Studies of the costs of inbred matings are now needed in similar species, such as naked mole-rats (*Heterocephalus glaber*), where captive females more commonly breed with close relatives, to determine whether these costs are lower than in Damaraland mole-rats.

## 1. Introduction

Mating between closely related individuals can result in a reduction in offspring fitness, known as inbreeding depression [1], and has been reported in both wild and captive populations across taxa [2,3]. The fitness costs associated with inbreeding may drive the selection of strategies that prevent individuals from breeding with close relatives, and inbreeding avoidance may shape patterns of reproduction and dispersal [4,5]. However, while many species avoid inbreeding with close relatives, some do not and may even preferentially mate with them [6], and it is predicted that inbreeding avoidance will only evolve when the chance of encountering mature relatives of the opposite sex is high and the effects of inbreeding depression are relatively strong [7].

In the social species of African mole-rats (*Bathyergidae*), a single unrelated pair usually monopolizes breeding in each group, and other adults rarely reproduce, with the result that levels of reproductive skew are among the highest seen in vertebrates [8]. Adults have unusually long lifespans [9,10], and their offspring of either sex commonly remain in their birth groups beyond reproductive age so that groups include a high frequency of siblings, which in the absence of incest avoidance, generates an elevated risk of inbreeding. In most social mole-rats, including Damaraland mole-rats (*Fukomys damarensis*), females avoid breeding with familiar males, and mating between close relatives does not occur [11,12]. While, in the naked mole-rat*,* inbreeding avoidance does not appear to be as extreme, and females will sometimes breed with close relatives in captive populations [13] though unrelated mating partners are preferred [14–16]. An experimental study of Mashona mole-rats (*Fukomys darlingi*) compared the survival of offspring born to pairs of cousins with those born to unrelated parents and showed a substantial reduction in the proportion of offspring weaned by related pairs [17]. As yet, despite the predicted importance of incest avoidance in maintaining reproductive skew [18], no studies have investigated the costs of inbreeding in the most social rodents, Damaraland and naked mole-rats, though analysis of a coronavirus epidemic in a captive population of naked mole-rats suggested that higher levels of mortality occurred in inbred individuals [19].

Here, we describe the consequences of inbreeding within sibling pairs in captive Damaraland mole-rats. Damaraland mole-rats live in stable groups of between two and 41 individuals including a single dominant breeding pair and their juvenile and adult offspring [20,21]. Females avoid mating with familiar, related males [22,23] and as subordinates rarely have access to an unfamiliar, unrelated male in their group, they seldom breed [24]. This analysis uses offspring born to both closely related breeding pairs and unrelated breeding pairs that were created for a previous experiment testing mechanisms of inbreeding avoidance in this species [23]. To induce breeding between closely related individuals, we cross-fostered litters of pups into unrelated breeding groups when they were approximately 10 days old. Once they reached adulthood, these individuals were paired with unfamiliar opposite-sex siblings (matched by age and weight) that had been raised in different groups. We subsequently measured and compared the fecundity of females mated to kin versus non-kin and the growth and survival of their pups. This experimental design allowed for a controlled comparison of offspring with variable inbreeding coefficients that were raised simultaneously in similar conditions.

## 2. Methods

### (a) Study animals and husbandry

All data were collected from a captive population of Damaraland mole-rats based at the Kuruman River Reserve in South Africa (26°58′ S, 21°49′ E). The population originated from 25 wild colonies (242 individuals) trapped near the research site between February and October 2013 and has since expanded through the formation of new colonies by pairing unrelated opposite-sex individuals. Groups were housed in standardized, self-contained tunnel systems of plastic pipe with transparent windows [25]. Animals were fed ad libitum on a diet of sweet potatoes and cucumbers and could be uniquely identified by a dye mark placed on their head and a passive integrated transponder tag injected subcutaneously.

Breeding groups were formed by placing two sexually mature individuals of opposite sex in a new tunnel system (full details are provided in [23]). Inbred groups were formed of sibling pairings where both parents were transferred to separate foster groups 10 days after birth and reared separately until pairing (eight groups, mean *r* = 0.44, ± 0.17 s.d.). Outbred pups were born to unrelated pairings (eight groups, mean *r* = −0.03, ± 0.12 s.d.) and reared separately in their birth groups. Calculations of genetic relatedness from previous analysis were used to confirm levels of relatedness between pairs (details can be found in [22] and the electronic supplementary material). All pairs successfully bred and continued breeding for a maximum of 53 months or until either breeder died. The mean reproductive tenure was 42 months for inbred groups (range 21–54 months) and 38 months for outbred groups (range 17–53 months).

### (b) Statistical analysis

All data analyses were performed in R v. 4.2.1 [26]. We modelled variation in litter size, interbirth interval and early-life survival between sibling and non-sibling pairings using linear mixed models (LMM) or generalized linear mixed models (GLMM), which were fitted using the *glmmTMB* package [27]. All model validation checks were done using the *Dharma* package [28]. We modelled longer-term survival using a Cox proportional hazards model containing both fixed and random effects fitted using the *coxme* package [29]. To model the growth of surviving individuals, we fitted a series of nonlinear mixed models (NLMMs) using the *nlme* package [30].

### (c) Litter size and interbirth intervals

Breeding females were checked for pregnancies every week by manual palpation and from weight increases that occur during gestation. As the estimated birthdate approached, we monitored females daily for signs of birth. After being born, pups were sexed and weighed to the nearest gram, and litter size was recorded. The litter size dataset contained 109 litters of known size (59 inbred and 50 outbred litters). The interbirth interval dataset contained an additional nine litters where pups were born but disappeared before they could be processed (so litter size was unknown), five from inbred pairs and four from outbred pairs, making a total of 118 breeding attempts. Interbirth intervals were defined as the number of days between litters.

Litter size was modelled to a Gaussian error distribution and interbirth interval to a gamma distribution (log-link). For both mixed effects models, we included treatment (inbred or outbred), group size and density as fixed effects, and maternal identity as a random effect. Density is measured as individuals in a colony per square metre of the tunnel and is included in all models alongside group size as tunnel systems are constrained by available lab space. For the interbirth interval model, we subtracted the estimated gestation length, conservatively taken as 90 days following the estimate of 78–92 days provided by Bennett & Faulkes [8] from the number of days between litters. This adjusted interbirth interval produced a distribution with a lower bound at 0 and a right skew that could be adequately handled by a gamma distribution.

### (d) Offspring birth weight

To analyse pup birth weights, we only considered pups that were weighed within 5 days of birth, a total of 292 pups (149 inbred and 143 outbred from 97 litters). Birth weight was modelled to a Gaussian error distribution with treatment, group size, litter size, density and age at weighing as fixed effects and maternal identity and litter identity as random effects.

### (e) Offspring survival

We analysed early-life survival in two steps. First, to test whether pup birth weight affected early-life survival, we modelled survival to 30 days using a GLMM with binomial error distribution and using the dataset of 292 individuals detailed above. Individual weight at birth, treatment, group size and density were included as fixed effects and maternal identity as a random effect. To test whether the effect of group size on offspring survival affected inbred and outbred individuals differently, we also fitted a model where we interacted with treatment with both group size and density. To investigate survival across development between treatments, we fitted a Cox proportional hazards model with maternal and litter identity included as random effects. To include pups that died very soon after birth and before they could be weighed, the latter two models used a larger dataset of 328 pups that were born between February 2020 and February 2024 (171 inbred and 157 outbred). One hundred and two individuals died from natural causes during the sampling period (mean age at death 51 days), and individuals that were removed from their natal groups for reasons other than death by natural causes (*n* = 82, mean age at removal 484 days; electronic supplementary material, table S2) were right censored in the survival analysis.

### (f) Inbreeding effects on growth

To test whether inbreeding affected growth, we compared the skeletal and body mass growth of inbred versus outbred individuals. Body mass was measured every week for the first three months of life and every two weeks thereafter, whereas the two skeletal measures, body length and upper incisor width, were only collected every six months as they were taken under anaesthesia (see electronic supplementary material). Incisor width is a repeatable measure of skull size and was measured at the widest point using digital callipers (± 0.1 mm). Body length was measured dorsally from the tip of the nose to the base of the tail using a tape measure (± 0.1 mm). Because of large sexual size dimorphism, we modelled the growth of males and females separately. A total of 4001 body mass measures were taken from 40 females (median = 104 per female), and 5125 measures were taken from 52 males (median = 102 per male). For the skeletal traits, we took 174 measurements from 39 females (median = 4 per female) and 217 measurements from 52 males (median = 4 per male). Previous work on captive Damaraland mole-rats has demonstrated that growth rates are fastest at birth and decelerate thereafter [31,32], so body mass and skeletal growth were modelled with either a monophasic or a biphasic monomolecular curve as detailed in the electronic supplementary material.

## 3. Results

### (a) Litter size and interbirth interval

The reproductive output of sibling breeding pairs did not differ from that of unrelated breeding pairs: there was no difference in litter size (LMM, estimate ± s.e. = 0.18 ± 0.34, *p* = 0.60; electronic supplementary material, table S3) between inbred (59 litters, mean = 2.89 pups, range = 1–6, s.d. = 1.28) and outbred (50 litters, mean = 3.14 pups, range = 1–6, s.d. = 1.10) groups or in interbirth interval (GLMM, estimate ± s.e. = 0.32 ± 0.28, *p* = 0.26; electronic supplementary material, table S4) between inbred (64 litters, mean = 142 days, range = 97–425 days, s.d. = 60) and outbred (54 litters, mean = 164 days, range = 94–818 days, s.d. = 114) groups.

### (b) Birth weight

**Figure 1.**
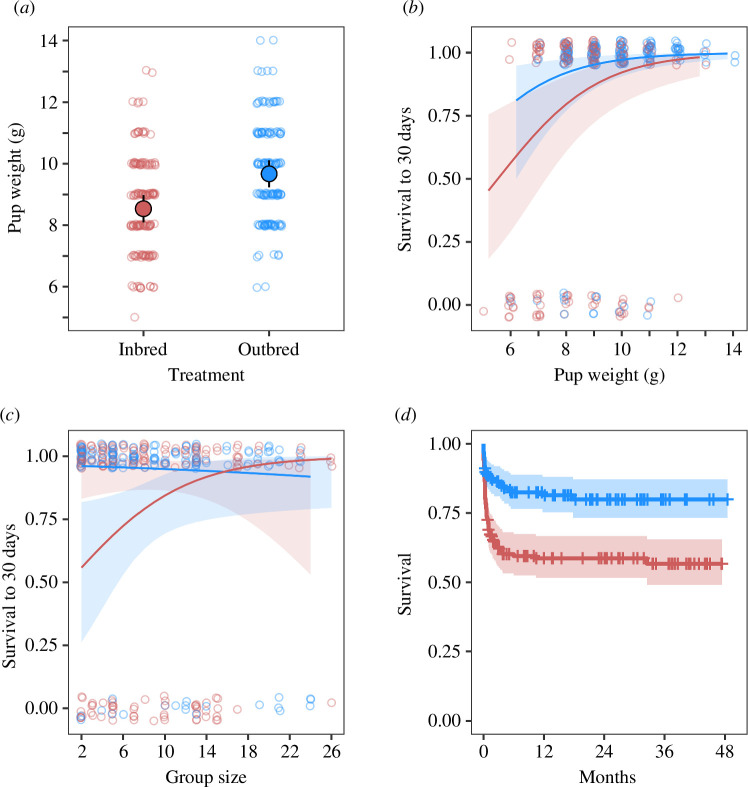
Inbred individuals show strong inbreeding depression for birth weight (*a*) and survival (*b*–*d*). All panels contrast individuals born to full sibling parents (red) with individuals born to unrelated parents (blue). Inbred individuals were lighter at birth (*a*), and low birth weights were associated with reduced survival to 30 days (*b*). The effects of inbreeding on offspring survival were strongest in small groups (*c*), and the reduced early-life survival of inbred pups carried over into adulthood (*d*). Plots (*a*–*c*) display the mean response (± 95% confidence intervals) predicted from mixed effects models alongside the raw data points, while plot (*d*) displays the Kaplan–Meier curve for inbred and outbred pups ± 95% confidence intervals.

Outbred pups (*n* = 143) were significantly (LMM, estimate ± s.e. = 1.13 ± 0.32, *p* ≤ 0.001; electronic supplementary material, table S5) heavier at birth than inbred pups (*n* = 149; by around 10%, see figure 1*a*), and pups born in larger groups were significantly lighter (LMM, estimate ± s.e. = −0.05 ± 0=.02, *p* = 0.02).

### (c) Offspring survival

Heavier birth weights were associated with an increased probability of pup survival to 30 days (GLMM, estimate ± s.e. = 0.55 ± 0.16, *p* ≤ 0.001; electronic supplementary material, table S6; figure 1*b*). As part of the effect of inbreeding on offspring survival was acting through pup weight, the effect of inbreeding was not significant when modelled alongside the pup weight (GLMM, estimate ± s.e. = 1.09 ± 0.65, *p* = 0.09). However, if pup weight was removed from the model, outbred pups showed significantly higher survival to 30 days (GLMM, estimate ± s.e. = 1.62 ± 0.64, *p* = 0.01). A significant interaction between group size and treatment indicated that for inbred pups, survival to 30 days was especially low in small groups (GLMM, estimate ± s.e. = −0.22 ± 0.10, *p* = 0.03; electronic supplementary material, table S7; figure 1*c*), whereas for outbred pups, survival was unrelated to group size.

Overall, 31% (102/328) of individuals died of natural causes during the sampling period. Deaths usually happened in the first few months of life, after which mortality rates were low (figure 1*d*). Individuals from inbred pairings showed lower survival than those from outbred pairings (Cox, estimate ± s.e. = −0.98 ± 0.42, *p* = 0.02; electronic supplementary material, table S8) as 41% (71/171) of inbred pups died during the sampling period compared with 20% (31/157) of outbred pups.

### (d) Skeletal and body mass growth

**Figure 2.**
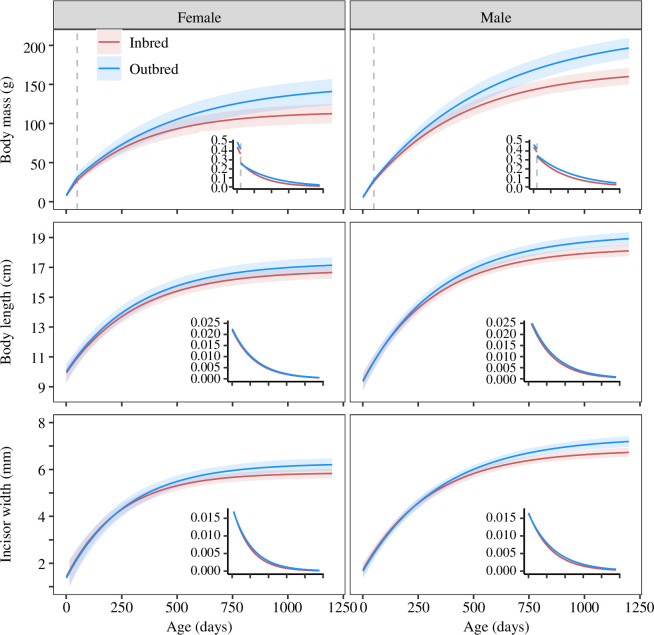
Effects of inbreeding on body mass growth and skeletal growth. Each panel displays the predicted growth trajectory for females and males (mean ± 95% confidence intervals) for body mass (upper), body length (middle) and upper incisor width (lower). Inset plots display the daily growth rates for each trait. The vertical dashed line in the body mass plots marks the threshold age at 50 days that separated the two phases of the biphasic curve and corresponds approximately with the age at weaning. The *x*-axis shows the individual age in days, and the scale is the same for all six panels and their inset plots.

Inbred individuals of both sexes (26 females and 27 males) grew more slowly than outbred individuals (14 females and 25 males) across development and reached a lower asymptotic body mass (NLMM, *A*_inb_ females = −36.06 ± 11.28, *t* = −3.20, *p* = 0.001; *A*_inb_ males = −50.56 ± 9.98, *t* = −5.06, *p* < 0.001; figure 2; electronic supplementary material, table S9). For females, inbreeding reduced asymptotic body mass by 23.9%, while for males, the effect was 23.0%.

For the skeletal traits of body length and incisor width, inbreeding was associated with significant reductions in the asymptotic size of males (27 inbred and 25 outbred; NLMM, *A*_inb_ body length = −0.92 ± 0.31, *t* = −2.96, *p* = 0.036 (electronic supplementary material, table S10); *A*_inb_ teeth width = −0.55 ± 0.17, *t* = −3.21, *p* = 0.002 (electronic supplementary material, table S11), reflecting a 4.9% reduction in body length and a 7.5% reduction in incisor width. Inbreeding was also associated with reductions in the asymptotic size of females (25 inbred and 14 outbred), but the effect did not reach significance for either trait (NLMM body length, *A*_inb_ = −0.52 ± 0.37, *t* = −1.40, *p* = 0.16; NLMM incisor width, *A*_inb_ = −0.366 ± 0.18, *t* = −1.97, *p* = 0.051).

## 4. Discussion

Our results provide evidence for inbreeding depression in Damaraland mole-rats across multiple traits. Inbred offspring were 10% lighter at birth and had a lower probability of early-life survival than offspring from unrelated parents. As adults, they were lighter in both sexes and had smaller skeletal measurements in males, where there was a 5% reduction in body length and 7.8% reduction in teeth width. Since individual differences in size and weight are correlated with breeding success in Damaraland mole-rats [33], the negative effect of inbreeding on early growth is predicted to result in reduced breeding success in adult life.

The negative effect of inbreeding on early-life survival disappeared in pups born into large groups. It is possible that this was due to the mitigating effect of non-breeding group members, who groom and retrieve young [25,34], keep young warm via huddling behaviour [35] and contribute to the creation of communal food stores that can be used by breeding females during lactation [36]. It has been shown in our captive population that increasing group size is associated with decreased workload in both breeders and non-breeders [37]. In cooperatively breeding species, the number of helpers is often correlated with breeding success [38,39], and the benefits of group living are predicted to play a key role in the evolution of mole-rat sociality [21,40], and though group size does not always bring strong fitness benefits in Damaraland mole-rats [41], it appears to have contributed to the improved survival of inbred pups in our study. In support of this argument, offspring care has been shown to buffer the effects of inbreeding depression in banded mongooses (*Mungos mungo*) [42] and some invertebrates [43,44]. Inbred individuals have also been shown to provide poor-quality care in banded mongooses [45] and some bird species [46,47]. Since inbred pups in our experiment were often raised in groups with inbred siblings, this might have contributed to the reduced survival of inbred pups in small to medium-sized groups.

While our study confirmed that inbreeding was associated with a reduction in offspring growth and survival, these costs were relatively low compared to those described by several previous studies of wild mammals. For example, in red deer (*Cervus elaphus*), highly inbred offspring had 77% lower first-year survival [48] although neonates from half-sibling parents were predicted to have a reduced birth weight of only around 4.4% [49]. This is not surprising, given that in captive populations, individuals may be partially shielded from the risk of predation, disease and parasitism. In addition, the availability of ad libitum food might help mitigate the effects of inbreeding on growth and survival, and several studies show that the severity of inbreeding depression is reduced in captive populations [50]. For example, experiments with deer mice (*Peromyscus leucopus noveboracensis*) showed that inbreeding costs in lab-reared animals released into natural populations were higher than those maintained in captive groups [51]. Similarly, studies show that stochasticity and harsh conditions can exacerbate the effects of inbreeding in wild populations [52,53].

While it was unsurprising that the costs of inbreeding in this study were low compared to those observed in wild mammals, the contrast in inbreeding costs between our study and a previous study of captive Mashona mole-rats [17] was surprising. In Mashona mole-rats, 76% of outbred pups survived to weaning while only 20% of inbred pups did so, while in our study, comparable survival rates (taken at a weaning age of one month [54]) were 87% for outbred pups and 69% for inbred pups. This difference is particularly surprising since in the Mashona mole-rat study, the inbred pups were first cousins (inbreeding coefficient = 0.125), whereas in our study, the parents of inbred pairs were full siblings (inbreeding coefficient = 0.25), so higher costs might be expected. It seems unlikely that this contrast is explained by differences in husbandry conditions, as the survival of outbred offspring was broadly similar between the two studies. Perhaps the clearest lesson to be drawn from this comparison is that levels of inbreeding depression may be affected by a range of environmental and genetic factors and can, therefore, vary in severity between species and across different ecological contexts. Given the importance of incest avoidance as a potential mechanism for mediating reproductive skew in social mole-rats [8], a prospective avenue of research is to explore inbreeding depression in naked mole-rats and determine whether it is lower than in this study, as the higher frequency of breeding between close relatives might suggest [13].

Finally, our results emphasize that inbreeding depression is unlikely to be confined to reductions in early-life traits such as birth weight and survival to independence. In addition, they may negatively affect lifetime traits that correlate with fitness, such as adult weight and skeletal size. These may lead to substantial reductions to the lifetime breeding success and fitness of the progeny of related breeding pairs, especially when environmental conditions are challenging.

## Data Availability

All data and code are available online from the Dryad Digital Repository [55]. Supplementary material is available online [56].
